# Unmasking Wnt-driven HCC: Inhibiting SMYD2 to overcome immune resistance

**DOI:** 10.1016/j.omton.2026.201298

**Published:** 2026-07-31

**Authors:** Xiupeng Chen, Allison M. Keeler, Joae Qiong Wu

**Affiliations:** 1 Horae Gene Therapy Center, Department of Genetic and Cellular Medicine, UMass Chan Medical School, Worcester, MA, USA; 2 Li Weibo Institute for Rare Diseases Research, UMass Chan Medical School, Worcester, MA, USA; 3 NeuroNexus Institute, UMass Chan Medical School, Worcester, MA, USA; 4 Department of Pediatrics, UMass Chan Medical School, Worcester, MA, USA; 5 Biochemistry and Molecular Biotechnology, UMass Chan Medical School, Worcester, MA, USA

## Main text

Hepatocellular carcinoma (HCC) remains one of the most lethal and therapeutically challenging malignancies worldwide, presenting a dismal prognosis for patients diagnosed at advanced stages.^1^ While immune checkpoint inhibitors (ICIs), particularly those targeting the programmed cell death 1 (PD-1)/PD-L1 axis, have significantly altered clinical oncology timelines, their efficacy in advanced HCC remains limited to a minority of patients.^2^ This modest response rate is primarily attributed to the highly heterogeneous and frequently immunosuppressive nature of the tumor microenvironment (TME), which is often characterized by T cell exclusion and myeloid infiltration.^3^ Consequently, identifying actionable epigenetic and molecular drivers that orchestrate this immune evasion is essential to developing rational, synergistic therapeutic combinations. In the previous issue of *Molecular Therapy Oncology*, Bueloni et al. provide critical insights into this therapeutic challenge by identifying the lysine methyltransferase SMYD2 as a potential regulator of oncogenic progression and immune exclusion in HCC, particularly in tumors with aberrant Wnt/β-catenin pathway activation.^3^

The study by Bueloni et al. sheds light on the role of SMYD2 in HCC by integrating transcriptomic analyses of patient samples with preclinical *in vivo* modeling, offering a conceptual link between tumor-intrinsic epigenetic hyperactivation and the extrinsic cellular reprogramming of the TME.^3^ Utilizing two structurally distinct SMYD2 inhibitors, LLY-507 *in vitro* and AZ-505 *in vivo*, the authors present evidence that pharmacological inhibition of SMYD2 correlates with suppressed tumor growth, phenotypic remodeling of the immunosuppressive microenvironment, and attenuation of Wnt/β-catenin-associated signaling, which collectively appear to sensitize Wnt-active HCC to anti-PD-1 immunotherapy.^3^

**Figure 1:**
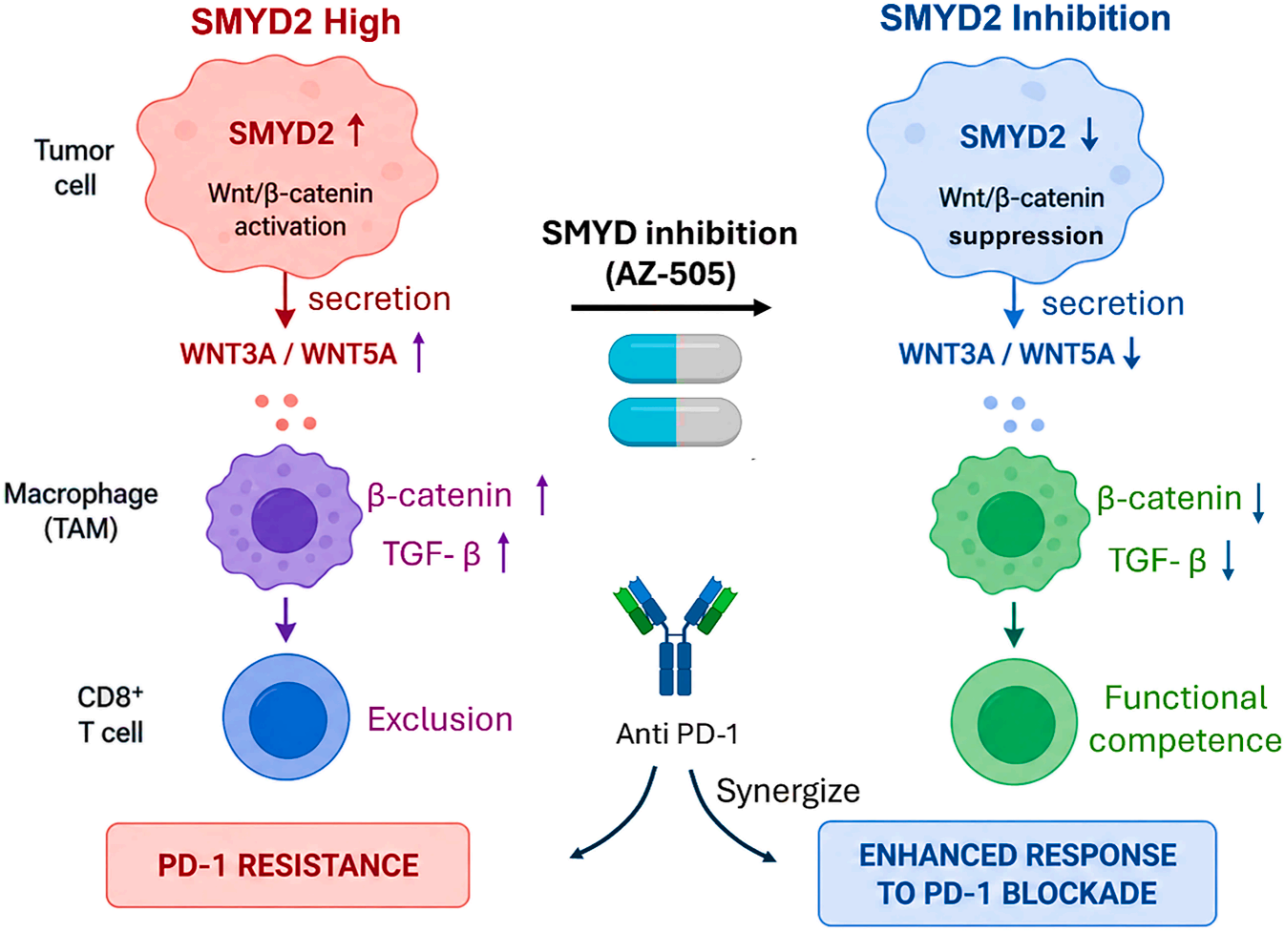
SMYD2 integrates oncogenic and immunosuppressive programs in hepatocellular carcinoma Schematic illustrating SMYD2-driven Wnt ligand production in tumor cells, activation of β-catenin signaling in macrophages, induction of TGF-β, and suppression of antitumor immunity. The figure also highlights the reversal of this axis by SMYD2 inhibition and synergy with PD-1 blockade.

Intriguingly, the implications of SMYD2 activity seem to extend beyond tumor-intrinsic programs to include a novel paracrine tumor-macrophage signaling axis. The study indicates that SMYD2 inhibition suppresses Wnt/β-catenin-associated transcriptional programs in tumor cells, accompanied by reduced expression of the Wnt ligands WNT3A and WNT5A (Figure 1).^3^ Conditioned-medium and pathway-modulation experiments further suggest that tumor-derived Wnt signals may promote Wnt/β-catenin activity in recipient macrophages, correlating with increased TGF-β expression. Disruption of this circuit via SMYD2 targeting appeared to attenuate macrophage-associated immunosuppressive signaling, thereby potentially remodeling the TME toward a more immune-permissive state.^4^ Importantly, the authors provide functional context showing that Wnt/β-catenin signaling in macrophages might actively contribute to sustaining an immunosuppressive phenotype rather than merely responding to tumor-derived cues. Using pharmacological modulation with DKK1 and CHIR99021, their data support a mechanistic link between tumor-derived Wnt signals and macrophage-mediated immune suppression, thereby broadening our understanding of how epigenetic regulators indirectly shape myeloid-driven networks.^4^ This paracrine model aligns with independent evidence that HCC-derived Wnt ligands drive M2-like polarization of tumor-associated macrophages through canonical Wnt/β-catenin signaling.

Beyond local effects on tumor-associated macrophages, the study highlights the broader immunological impact of SMYD2 inhibition in orthotopic HCC models. Notably, anti-PD-1 monotherapy and the combination treatment induced comparable increases in intratumoral CD8^+^ T cell abundance. Therefore, the superior efficacy of the combination could not be explained by additional intratumoral CD8^+^ T cell accumulation. Instead, only the combination of AZ-505 and anti-PD-1 elicited systemic immune activation, as suggested by elevated splenic CD8^+^ T cell frequencies, enhanced antigen-presenting cell activation, and increased *ex vivo* CD8^+^ T cell degranulation capacity.^3^ This apparent emphasis on peripheral activation versus intratumoral recruitment suggests that SMYD2 inhibition may improve the functionality of anti-tumor immune responses without necessarily requiring extensive additional T cell infiltration into the core tumor microenvironment.

From a translational perspective, a compelling aspect of this study is the synergistic antitumor activity observed with SMYD2 inhibition and PD-1 blockade in Wnt/β-catenin-active HCC, a molecular subtype frequently associated with primary resistance to immunotherapy.^5^ In this context, combination treatment induced comprehensive immune transcriptional remodeling characterized by predicted activation of IL-12 and IFNγ pathways alongside Th1 polarization, while concurrently suppressing pathways linked to IL-10, STAT3, and alternative macrophage activation. Biologically, these findings broaden the functional scope of SMYD2 beyond its established roles in tumor cell proliferation, positioning it at the interface of epigenetic regulation and cancer immunity.^6^ Furthermore, the data suggest a context-dependent therapeutic vulnerability, as Wnt/β-catenin-active, TP53-wild-type tumors appeared particularly responsive to SMYD2 inhibition.^3^ This observation is consistent with the distinct molecular subclasses that characterize HCC, raising the possibility that specific genomic backgrounds dictate SMYD2 dependency.^7^

While this study provides a comprehensive framework linking SMYD2 to tumor-intrinsic signaling and TME modulation, it also opens several avenues for future exploration. First, although the data support a role for SMYD2 in modulating β-catenin-associated transcriptional programs, the precise molecular intermediates require further elucidation at the biochemistry level. SMYD2 acts on both histone and non-histone substrates.^6^ Therefore, future studies are needed to determine whether additional chromatin-level or co-regulatory mechanisms contribute to the observed suppression of Wnt/β-catenin signaling, beyond the previously reported effects of β-catenin methylation and the reduced Wnt-ligand expression observed in the present study. One intermediate candidate is SMYD2-mediated methylation and stabilization of c-Myc, a shared driver oncogene in both murine and human tumor models.^8^ SMYD2 inhibition may also act through a complementary, tumor-intrinsic route, derepressing DNA-damage signaling to activate cGAS-STING-dependent antitumor immunity.^9^ Second, patient transcriptomic datasets consistently link SMYD2 expression to Wnt/β-catenin-driven immunosuppressive states. Confirming how this axis is anatomically organized within the human TME will require validation using spatially resolved or single-cell approaches from human HCC samples. Third, dissecting how SMYD2-targeted interventions reshape systemic versus tissue-resident immune compartments will be important for optimizing combination strategies with ICIs.

In conclusion, Bueloni et al. present a provocative rationale for targeting SMYD2 to modulate the immune microenvironment in Wnt/β-catenin-active HCC.^3^ By linking epigenetic regulation to myeloid-driven T cell exclusion, their preclinical evidence provides a strong foundation for future studies investigating SMYD2 not only as a therapeutic target but also potentially as a biomarker for molecular stratification in combination immunotherapy strategies. Notably, the broader strategy of combining epigenetic enzyme inhibitors with checkpoint blockade has already entered early-phase clinical testing for immunotherapy-refractory HCC,^10^ although SMYD2-selective inhibitors such as AZ-505 and LLY-507 currently are only used preclinically; therefore, further medicinal chemistry and safety development will be required before clinical approval or translation.

## Declaration of interests

The authors declare no conflicts of interest related to this commentary.
